# Evaluating Long-Term Outcomes of a High School-Based Impaired and Distracted Driving Prevention Program

**DOI:** 10.3390/healthcare10030474

**Published:** 2022-03-03

**Authors:** Lindsay Buczek, Laura K. Gryder, Samantha Slinkard-Barnum, Kavita Batra, Cassandra Trummel, Allison G. McNickle, Douglas R. Fraser, Deborah A. Kuhls, Paul J. Chestovich

**Affiliations:** 1 Kirk Kerkorian School of Medicine at University of Nevada, Las Vegas, NV 89102, USA; buczek@unlv.nevada.edu; 2 Department of Surgery, Kirk Kerkorian School of Medicine at University of Nevada, Las Vegas, NV 89102, USA; allison.mcnickle@unlv.edu (A.G.M.); douglas.fraser@unlv.edu (D.R.F.); deborah.kuhls@unlv.edu (D.A.K.); paul.chestovich@unlv.edu (P.J.C.); 3 Center for Rural and Primary Healthcare, University of South Carolina School of Medicine, Columbia, SC 29209, USA; samantha.slinkard-barnum@uscmed.sc.edu; 4 Office of Research, Kirk Kerkorian School of Medicine at University of Nevada, Las Vegas, NV 89102, USA; kavita.batra@unlv.edu; 5 Southwest Career and Technical Academy, Las Vegas, NV 89113, USA; trummc@nv.ccsd.net

**Keywords:** impaired driving, distracted driving, teen drivers, driver safety, injury prevention, motor vehicle crash, educational program

## Abstract

Motor vehicle crashes are one of the leading causes of death among teenagers. Many of these deaths are due to preventable causes, including impaired and distracted driving. You Drink, You Drive, You Lose (YDYDYL) is a prevention program to educate high school students about the consequences of impaired and distracted driving. YDYDYL was conducted at a public high school in Southern Nevada in March 2020. A secondary data analysis was conducted to compare knowledge and attitudes of previous participants with first-time participants. Independent-samples-*t* test and χ^2^ test/Fisher’s exact test with post-contingency analysis were used to compare pre-event responses between students who had attended the program one year prior and students who had not. Significance was set at *p* < 0.05. A total of 349 students participated in the survey and were included for analysis; 177 had attended the program previously (50.7%) and 172 had not (49.3%). The mean age of previous participants and first-time participants was 16.2 (SD ± 1.06 years) and 14.9 (SD ± 0.92 years), respectively. Statistically significant differences in several self-reported baseline behaviors and attitudinal responses were found between the two groups; for example, 47.4% of previous participants compared to 29.4% of first-time participants disagreed that reading text messages only at a stop light was acceptable. Students were also asked how likely they were to intervene if a friend or family member was practicing unsafe driving behaviors; responses were similar between the two groups. The baseline behaviors and attitudes of participants regarding impaired and distracted driving were more protective among previous participants compared to first-time participants, suggesting the program results in long-term positive changes in behaviors and attitudes. The results of this secondary retrospective study may be useful for informing the implementation of future impaired and distracted driving prevention programs.

## 1. Introduction

Over 39,000 people were killed following motor vehicle crashes in the United States (U.S.) in 2018, of which nearly seven percent were young drivers (13–19 years old) [1]. Reportedly, motor vehicle traffic injury was one of the leading causes of premature mortality among young drivers from 1999 to 2016 [2]. Particularly, impaired (under the influence of drugs and/or alcohol) and distracted driving among young drivers is attributed to a significant proportion of road fatalities. 

In 2019, over 1300 young drivers under 20 years of age died in a motor vehicle crash; one in five had a blood alcohol concentration (BAC) over 0.08 percent [3]. While it is legal for drivers 21 years and older to have a BAC less than 0.08 percent in most states, drivers younger than 21 years old are subject to zero tolerance laws and are not permitted to have any alcohol in their system [4,5]. The odds of a crash increase while driving under the influence of alcohol due to its impairing effects on an individual’s ability to drive [6]. According to the Insurance Institute for Highway Safety (IIHS), young drivers aged 16–20 with a BAC of 0.08 percent were ten times more likely to be involved in a fatal crash than those who had not consumed alcohol [6]. Teens driving under the influence of drugs is a grave concern, as many drugs can impair an individual’s ability to focus, coordinate, and react [7]. According to the recent nationally representative (U.S.) Youth Risk Behavior Surveys (YRBS), students who reported using marijuana were more likely to engage in risky driving behaviors, including failure to obey traffic laws and distracted driving [8]. 

Distracted driving, which is typically due to visual, manual, or cognitive distractions, can significantly increase the risk of a motor vehicle crash [9,10]. Results from the Second Strategic Highway Research Program Naturalistic Driving Study indicated that visual-manual tasks, such as texting while driving, created an increased risk for drivers of all ages; however, distractions of all types were found to be more prevalent among drivers under 20 years of age [11]. In 2015, seven percent of all fatal crashes in the U.S. involved young drivers (age 15–19), with teens comprising 3.9 percent of all licensed drivers during this same period [12,13]. In addition, nine percent of the fatal crashes involving teen drivers involved distracted driving [13]. Texting while driving has a 23-times increased risk of crash [9]. In the 2017 YRBS survey, nearly 39 percent of students reported that they had sent a text or email while driving in the 30 days prior to the survey [14]. Using electronic cigarettes, or vaping, can also be a form of manual distraction [10]. The overall prevalence of nicotine vaping (not limited to only vaping while driving) has increased among twelfth grade students from 18.8 percent in 2017 to 35.1 percent in 2019 [15]. These figures are concerning and underscore the need for targeted prevention and intervention programs that promote safe driving behaviors among vulnerable groups, i.e., young drivers.

Several programs have been designed and instituted to promote safe driving practices among young drivers and to reduce motor vehicle crashes attributed to impaired and distracted driving [16,17,18,19,20,21]. These programs differ in their effectiveness to achieve desired short-term and long-term goals. Despite the large amount of literature available regarding driving safety programs, there is a paucity of literature that evaluates the sustainment of knowledge and safe driving behaviors. The assessment of sustainment of knowledge is of particular importance, as this provides further information on the long-term effectiveness of impaired and distracted driving prevention programs. Therefore, this study aims to determine differences in self-reported behaviors and attitudes between high school students who had previously participated in an impaired and distracted driving prevention program called You Drink, You Drive, You Lose (YDYDYL) and first-time participants via a secondary retrospective study.

## 2. Materials and Methods

### 2.1. Program Description

**Figure 1:**
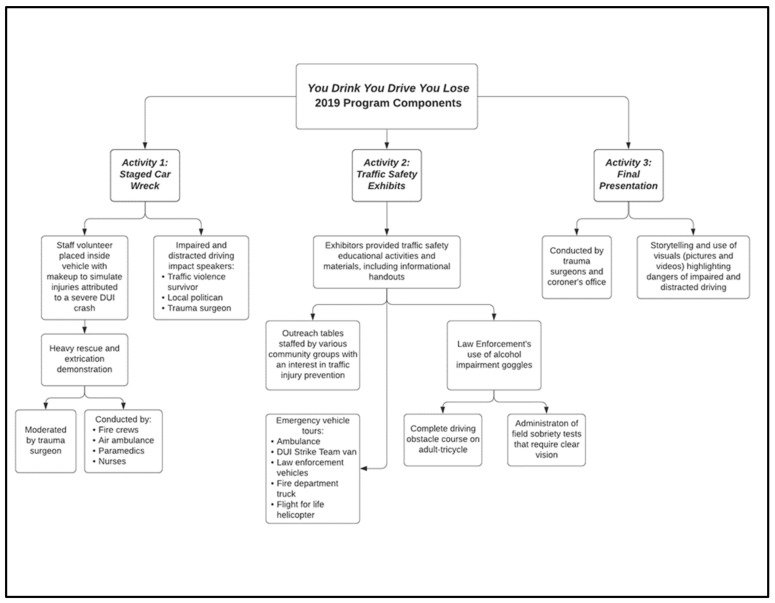
You Drink, You Drive, You Lose (YDYDYL) 2019 Program Components.

YDYDYL is a program that aims to educate young drivers about the dangers of impaired and distracted driving in order to promote safe driving behaviors. YDYDYL was offered to students in March of 2019 and 2020 at a public high school located in Southern Nevada by injury prevention specialists from a local Level I trauma center. The risks and consequences of impaired and distracted driving are taught to students through community collaboration, interactive practical activities, and heavy rescue demonstrations. Behavioral outcomes were assessed via survey. The program is a collaborative effort of several groups, including internal and external traffic safety and injury prevention partners, ambulance companies, fire departments, hospitals, university groups, funeral services, county coroner’s offices, local law enforcement agencies (including county school district police and a regional multi-jurisdictional Driving Under the Influence [DUI] “strike team”), organ donation organizations, and other traffic safety community groups. A detailed description of the 2019 program elements is provided in Figure 1.

YDYDYL has been administered to high school students at a Southern Nevada Level I trauma center for over two decades. In 2019 the program was adapted to be held at a local high school (grades 9–12), with program activities occurring during the first half of the school day. This adaptation expanded the reach of safety messaging to a larger audience, increasing from approximately 150 to 400 students exposed in the new program format. The 2020 program was held at the same high school as the 2019 program. Participation was once again offered to all students of grades 9–12. The program was administered in the same format as in 2019, with slight modifications based on 2019 student survey feedback.

### 2.2. Study Type and Data Source

This evaluation is a secondary retrospective study. The goal of the study was to evaluate the YDYDYL program using data that had previously been collected from participants by the program administrators for the purposes of internal evaluation. The 2020 survey was administered to student attendees two days prior to the event via an anonymous Qualtrics survey link distributed by the YDYDYL program planners. An algorithm (called Prevent Ballot-box Stuffing) in the survey platform Qualtrics was used to prevent multiple responses from the same participants. In addition, each student was required to create a unique identifier to prevent duplication. Questions related to previous participation in YDYDYL, demographic information, impaired and distracted driving behaviors and attitudes were included in the survey administered by the program. The questionnaire was adapted from a pre-existing survey which was used to assess a different teen impaired driving prevention program called Every 15 Min [16].

### 2.3. Ethical Considerations

There was no direct involvement of human subjects in this evaluation of YDYDYL. The data used in this study were previously collected via anonymous surveys administered by the program planners. Due to the secondary retrospective design of this program evaluation, the University of Nevada, Las Vegas School of Medicine Institutional Review Board determined this research project to be exempt.

### 2.4. Sample Size Justification

Sample size determination was done through G*Power software using Cohen’s medium effect size of 0.3, α = 0.05, and a desired power of 80% for a chi-square test [22,23]. The minimum sample required was 143 and after factoring 10% missing data, the final sample size required was 157, which was comparable to our sample size.

### 2.5. Statistical Analysis

Participants’ responses were first preprocessed or cleaned and then exported to IBM SPSS version 26.0 (IBM Corp. Armonk, NY, USA) for statistical analyses. Incomplete responses and those with invalid data entries were excluded. Mean and standard deviation were used to represent continuous variables. Counts and proportions were used to express categorical variables. Inferential statistics were conducted through Chi-square/Fisher's-exact test, and Independent samples-t-tests were used to perform group comparisons. The normality and homoscedasticity assumptions were assessed through the Shapiro-Wilk test and F-tests. All analyses were conducted at α = 0.05. For statistical reporting, a Checklist for Statistical Assessment of Medical Papers (CHAMP statement) was utilized [24].

## 3. Results

**Figure 2:**
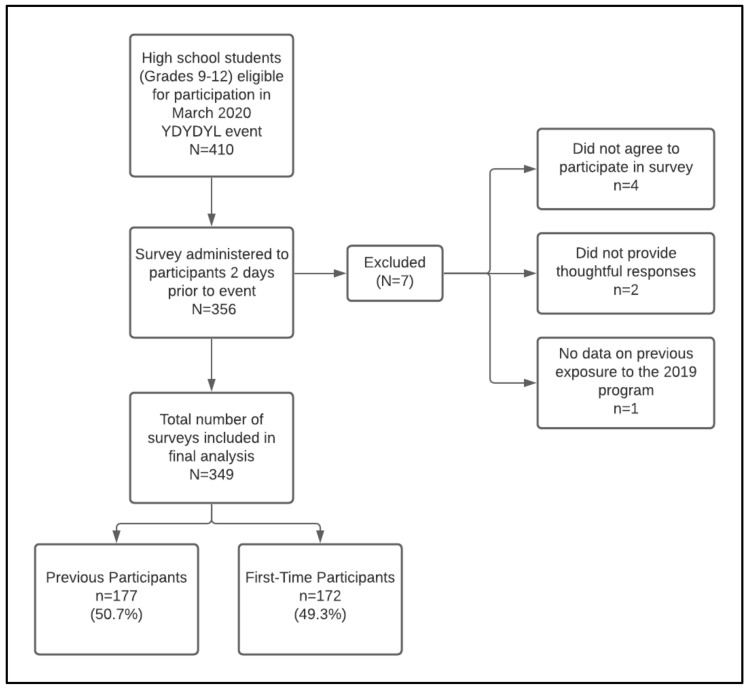
You Drink, You Drive, You Lose (YDYDYL) 2020 event survey flow-diagram.

A total of 410 high school students were invited to participate in the program, of which 356 (86.8%) completed the survey, and 349 (85.1%) were included for analysis (Figure 2). Those who did not participate were either absent or chose not to attend the event due to personal reasons. Among the students who participated in the survey, 177 (50.7%) were previous participants in the program and 172 (49.3%) students were first-time participants. previous participants (mean ± SD = 16.2 ± 1.06 years old) were older than first-time participants (mean ± SD = 14.9 ± 0.92 years old) (*p* < 0.001). In addition, 57.1% of previous participants had a driver’s license or learner’s permit compared to only 15.1% among first-time participants (*p* < 0.001). The demographic profile of first-time participants and previous participants was comparable, which is shown in Table 1. 

As indicated in Table 2, the two participant groups did not significantly differ in terms of history of using impairing substances and there was no significant difference in proportions of participants who had ever tried marijuana between groups (previous participants = 10.2% vs. first-time participants = 7.6%, *p* = 0.401). As indicated in Table 3, nearly 8% of first-time participants had driven while “buzzed” or drunk while no previous participant drivers reported this behavior. 

**Figure 3:**
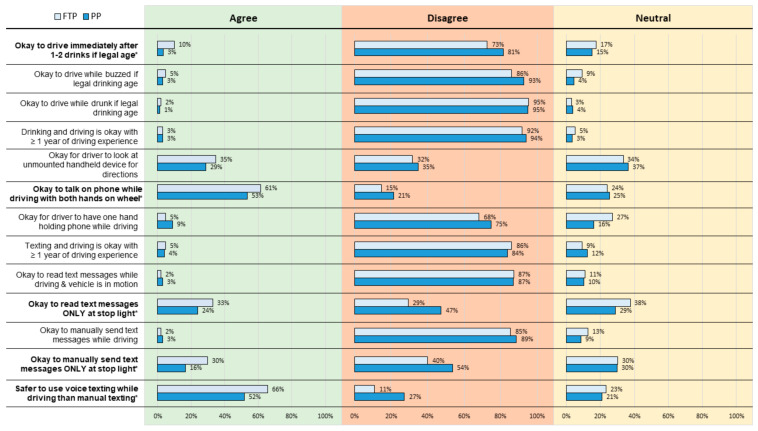
Attitudes of driving behaviors among first-time participants (FTP) and previous participants (PP). Statements in bold * are statistically significant. *p*-values < 0.05 are statistically significant. Note: “Agree” and “Strongly Agree” responses were combined; “Disagree” and “Strongly Disagree” were also combined. The percentages shown in the figure are rounded up to the nearest integer.

In addition to behavioral responses, statistically significant differences were found in attitudes towards impaired and distracted driving between the two groups (Figure 3). When asked if it is “okay” to drive immediately after consuming 1–2 alcoholic beverages by a driver who is of legal drinking age, a higher proportion of previous participants disagreed with the statement (81.5% vs. 72.7%) in comparison to first-time participants (*p* = 0.023). When asked if it is “okay” to talk on the phone while driving with both hands on the wheel (i.e., use of a hands-free phone device), both groups agreed with the statement; first-time participants agreed more strongly (61.4% vs. 53.3%) in comparison to previous participants (*p* = 0.043). A significant difference in attitudes was also found in responses to the statement that it is “okay” to read text messages only at a stop light; previous participants disagreed (47.4%) compared to only 29.4% of first-time participants (*p* < 0.001). A difference in group attitudes was noted when asked a similar question about manually sending text messages only when at a stop light; the majority (53.7%) of previous participants and 40.1% of first-time participants disagreed, conveying that this behavior is not acceptable (*p* < 0.001). Regarding attitudes toward the statement that it is safer to use voice texting while driving versus manual texting, previous participants and first-time participants both agreed (51.7%, vs. 65.7%); however, previous participants' average scores were closer to neutral than first-time participants, wherein more first-time participants believe that voice texting while driving is safer than manual texting (*p* < 0.001). When asked how likely they were to intervene if a friend was driving while intoxicated from alcohol, 92.3% of previous participants and 89.3% of first-time participants reported that they were likely to intervene (*p* < 0.001, Table 4). Similarly, 88.2% of previous participants and 89.6% of first-time participants reported that they would be likely to intervene if a family member was driving while intoxicated from alcohol (*p* < 0.001). The majority of previous participants (86.0%) and first-time participants (83.8%) also reported that they would likely intervene if a friend was driving 30 min after drinking two alcoholic beverages (*p* = 0.03); similar findings were observed if the driver was a family member of the previous participants (86.5%) and first-time participants (82.0%) (*p* = 0.01). 

## 4. Discussion

The most interesting aspect of this study was sustainment of self-reported safe driving behaviors among former participants, which is vital for honing safe driving habits among young drivers who are more susceptible to engaging in risky behavior due to myriad developmental and social factors [25]. Given that behavior patterns emerging in early adolescence influence behaviors in adulthood, programs with favorable long-term behavioral outcomes would be of paramount significance [25]. Compared to other traffic safety behavioral programs, such as Every 15 Minutes [16] and You Hold the Key [17], one main strength of this assessment of the YDYDYL program is the measurement of long-term attitudinal and self-reported behavioral changes. Many programs with a few exceptions (e.g., alcohol misuse prevention program) only measure short-term outcomes [18] for practical process-related reasons; however, long-term assessment of programs is critical in strengthening the evidence of efficacy, which the current study sought to provide. This analysis of long-term outcomes following implementation of the YDYDYL program indicates that the previous participants had more protective self-reported behaviors and safe driving attitudes than the first-time participants. These findings lend support to the implementation of targeted interventions focusing on the education of young drivers to promote safer driving habits. Programs such as YDYDYL can assist young drivers in establishing protective lifelong driving behaviors, as the program is offered to students at a time when they are learning how to drive and do not yet have much experience. In addition, exposing students to the dangers of impaired and distracted driving at a young age may help decrease the overall incidence of crashes due to these causes.

Despite these results, there were no statistically significant attitudinal differences between the two groups regarding other impaired and distracted driving risk-taking behaviors. An example of one such behavior includes talking on the phone with both hands on the wheel while driving. While previous participants of the program one year prior learned that any distraction behind the wheel is dangerous (hands-free or not), their attitudes may also be influenced by having significantly more driving experience in comparison to first-time participants, wherein some risk-taking behaviors may have been normalized with greater exposure to driving. In addition, driving while using a hands-free device is legal in Nevada, therefore participants of both groups may believe that the behavior is safe. Finally, previous participants and first-time participants may have reported similar attitudes where actions are widely accepted to be dangerous and unacceptable (i.e., driving immediately after consuming alcohol). 

Our findings in this secondary retrospective study related to the likelihood of stopping friends/family members from engaging in dangerous driving behaviors reveal that over 80% of previous participants (Table 4) and first-time participants (Table 5) would intervene. For example, previous participants were likely to stop a friend from driving 30 min after consuming two alcoholic drinks (86.0%), as were first-time participants (83.8%). These results point to the importance of developing peer-to-peer educational interventions to encourage students who were not likely or felt neutral about intervening in these hypothetical situations. The YDYDYL program may benefit from the addition of a peer-mentoring model to further promote the acquisition of safe driving habits among teens. In an evaluation of Learn2Live, a risky driving prevention program targeted at young drivers and passengers, it was found that program participants favored the event held by peers rather than the event held by various experts [19]. However, there were no significant differences found in the reported attitudes between the two groups in Learn2Live [19]. Similarly, the Students Against Destructive Decisions peer organization was not found to significantly reduce students’ unsafe driving practices (i.e., driving after drinking alcohol and/or riding with a driver who had been drinking alcohol) [20]. It is possible that peer-mentoring may encourage students to take a more active role in speaking up to prevent friends and family members from driving while under the influence. In addition, the literature shows that having a designated portion of the program in which students learn the skills needed to speak up to friends and family can make students more likely to intervene and potentially prevent a dangerous situation from occurring [21]. These findings can be applied to other programs in Nevada as well as across the U.S. to further promote safe driving behaviors. 

### Strengths and Limitations

This study is the first one (to our knowledge) to determine the long-term effect of the YDYDYL program. Therefore, the results from this study could be used to inform programs in other regions and may aid in fostering sustainable long-term outcomes such as more protective driving habits. Like other studies, this study is not without limitations. First, due to geographical restriction (i.e., Southern Nevada-based), the external validity will be questionable, as findings of this study may not be extrapolated to other parts of the nation. Second, the cross-sectional research design does not allow for causal inferencing. A more powerful research design, such as a randomized controlled trial (RCT) can be used to study the effectiveness of an intervention on the outcome variable while reducing various forms of bias, however RCTs are not always possible or practical. The YDYDYL program was evaluated at a single school in which all students were offered the opportunity to attend the program. The surveys were not distributed to another school that did not participate in the program, so it was not possible to compare the responses of a control school with the participating school. Third, while the results suggest that students formed safer driving habits as a result of being exposed to YDYDYL, there are additional factors that could have influenced the outcomes. Factors such as the history of family members involved in motor vehicle crashes, past experiences of family/friends, and previous engagements in other safe driving programs were not measured, which may contribute to residual confounding. In other words, one cannot infer that positive outcomes are exclusively associated with the program. It is likely that negative events such as the death of friends and/or family in a car crash and past experiences may impact students’ driving behaviors and attitudes, contributing to the adoption of safe habits by some participants compared to their classmates. Fourth, maturation could confound internal validity in that the differences observed between these two groups may have occurred as a function of the passage of time regardless of exposure to the program. Therefore, our finding that previous participants reported safer behaviors and attitudes may be due to the program, student age, personal experience, or a combination of factors. The social desirability bias might have affected the results, as data were based on self-reported information and participants may have been less likely to reveal risky behaviors. In addition, reported behaviors may vary from actual behaviors due to a variety of reasons, including the herd effect (i.e., group mind), wishful thinking (i.e., the Lake Wobegon effect), and differing contexts (external or internal). It is nearly impossible to predict how individuals will behave in a different context or in the real world [26,27,28]. Lastly, the questionnaire implemented by the program was only available in English, which may have excluded non-English speakers from participating in the survey. 

## 5. Conclusions

The You Drink, You Drive, You Lose (YDYDYL) program had promising results related to sustainment of safe driving behaviors. Previous participants of the program initiated and sustained protective behaviors as compared to the first-time participants. While the majority of participants in both groups reported their intent to persuade their family/friends to quit unsafe driving practices, there were no statistically significant differences measured between groups. This underscores the need to develop a concerted, consistent approach utilizing a peer-mentoring component.

### Future Research Directions

The findings of this study may serve as baseline data to develop and refine impaired and distracted driving prevention programs at broader levels. In the future, similar programs can be developed to include or focus their message to other demographics (e.g., age, race/ethnicity, socioeconomic status, urbanization level, road user type, etc.). This program is not unique to Southern Nevada and has previously been implemented by organizations in other geographical regions, however its effectiveness in other regions still needs to be measured to strengthen existing evidence presented by the findings of this study. In addition, studies comparing multiple years of data can be planned to see if long-term benefits of the program persist for future cohorts of program attendees. Future study designs using the surveys with greater validity evidence grounded in robust theoretical frameworks would be beneficial for the application of advanced statistical modeling techniques. This will bridge the gap between theory and observation for developing evidence-based interventions.

## Figures and Tables

**Table 1:** Demographic Characteristics of Previous and First-time Participants (*n* = 349).

	Previous Participants (*n* = 177, 50.7%)	First-time Participants (*n* = 172, 49.3%)	Test Statistics *p*-Value
	*n* (%)	*n* (%)	Chi-Square *p*-Value
Gender:			*Χ*^2^ = 5.30 *p* = 0.151
Male	85 (48.0)	94 (54.7)	*Χ*^2^ = 1.19 *p* = 0.236
Female	87 (49.2)	75 (43.6)	*Χ*^2^ = 1.09 *p* = 0.276
Non-binary	3 (1.7)	0 (0.0)	*Χ*^2^ = 1.72 *p* = 0.085
Prefer not to say	1 (0.6)	3 (1.7)	*Χ*^2^ = 1.03 *p* = 0.303
Race/Ethnicity:			*Χ*^2^ = 0.76 *p* = 0.682
White	63 (35.6)	60 (34.9)	*Χ*^2^ = 0.10 *p* = 0.921
Non-White *	75 (42.4)	79 (45.9)	*Χ*^2^ = 0.72 *p* = 0.473
Hispanic or Latino	39 (22.0)	32 (18.6)	*Χ*^2^ = 0.77 *p* = 0.442
Licensure Status:			*Χ*^2^ = 66.40 *p* ≤ 0.001
Driver’s license	47 (26.6)	11 (6.4)	*Χ*^2^ = 5.06 *p* ≤ 0.001
Learner’s permit	54 (30.5)	15 (8.7)	*Χ*^2^ = 5.11 *p* ≤ 0.001
Neither	76 (42.9)	146 (84.9)	*Χ*^2^ = 8.14 *p* ≤ 0.001

Χ^2^ = Chi-square; *p*-values < 0.05 are statistically significant; * Non-White group includes American Indian or Alaskan Native, Asian, Black or African American, Native Hawaiian or Another Pacific Islander, Mixed or Multiracial, and Other. Some categories variables are not mutually exclusive therefore percentages may exceed 100%.

**Table 2:** Baseline Behavioral Responses (*n* = 349).

Survey Questions	Previous Participants (*n* = 177)	First-Time Participants (*n* = 172)	Chi-Square *p*-Value
	Yes *n* (%)	Yes *n* (%)	
Have ever tried:	177	172	
Alcohol	52 (29.4)	41 (23.8)	*Χ*^2^ = 1.37 *p =* 0.242
Marijuana	18 (10.2)	13 (7.6)	*Χ*^2^ = 0.71 *p* = 0.401
Vaping (any substance)	19 (10.7)	19 (11.0)	*Χ*^2^ = 0.01 *p =* 0.925
Other Illegal substances	4 (2.3)	4 (2.3)	*Χ*^2^ = 0.00 *p =* 0.622 *
Ridden in a vehicle driven by someone who had been drinking alcohol	58 (32.8)	43 (25.0)	*Χ*^2^ = 2.56 *p =* 0.110
≤30 days, rode in a vehicle with a driver who had been drinking alcohol	14 (7.9)	18 (10.5)	*Χ*^2^ = 3.58 *p =* 0.06

Χ^2^ = Chi-square; *p*-values < 0.05 are statistically significant; * Generated from Fisher’s exact test.

**Table 3:** Driver Baseline Behavioral Responses (*n* = 127).

Survey Questions	Previous Participants (*n* = 101)	First-Time Participants (*n* = 26)	Chi-Square *p*-Value
	Yes *n* (%)	Yes *n* (%)	
Drivers who have ever driven			
Buzzed/drunk	0 (0.0)	2 (7.7)	*Χ*^2^ = 7.82 *p* = 0.041 *
After using marijuana	1 (1.0)	1 (3.8)	*Χ*^2^ = 1.14 *p* = 0.361 *
While vaping any substance	6 (5.9)	1 (3.8)	*Χ*^2^ = 0.19 *p* = 0.662
≤30 days, driven while buzzed/drunk	0 (0.0)	1 (3.8)	*Χ*^2^ = 3.80 *p* = 0.210 *
≤30 days, driven after using marijuana	0 (0.0)	1 (3.8)	*Χ*^2^ = 3.91 *p* = 0.205 *
≤30 days, driven while vaping (any substance)	1 (1.0)	1 (3.8)	*Χ*^2^ = 1.03 *p* = 0.377 *
≤7 days, sent text message(s) while driving &vehicle was in motion	30 (29.7)	7 (26.9)	*Χ*^2^ = 0.08 *p* = 0.781

Χ^2^ = Chi-square; *p*-values < 0.05 are statistically significant; * Generated from Fisher’s exact test.

**Table 4:** Previous participants likelihood of stopping a friend or family member practicing unsafe driving behaviors (*n* = 177).

Survey Questions	Likely	Neutral	Not-Likely
	*n* (%)	*n* (%)	*n* (%)
How likely you would stop a **friend** from practicing the following behaviors			
Driving when they are intoxicated from alcohol	159 (89.3)	8 (4.5)	11 (6.2)
Driving 30 min after consuming two alcoholic drinks	153 (86.0)	11 (6.2)	14 (7.9)
Driving after using marijuana	148 (83.6)	14 (7.9)	15 (8.5)
Driving after using illegal drugs	156 (88.6)	6 (3.4)	14 (8.0)
How likely you would stop a **family member** from practicing the following behaviors			
Driving when they are intoxicated from alcohol	157 (88.2)	11 (6.2)	10 (5.6)
Driving 30 min after consuming two alcoholic drinks	154 (86.5)	12 (6.7)	12 (6.7)
Driving after using marijuana	150 (84.3)	14 (7.9)	14 (7.9)
Driving after using illegal drugs	159 (89.3)	8 (4.5)	11 (6.2)

Note: “Likely” and “Very Likely” responses were combined; “Very Unlikely” and “Not-Likely” were also combined.

**Table 5:** First-time participants likelihood of stopping a friend or family member practicing unsafe driving behaviors (*n* = 172).

Survey Questions	Likely	Neutral	Not-Likely
	*n* (%)	*n* (%)	*n* (%)
How likely you would stop a **friend** from practicing the following behaviors			
Driving when they are intoxicated from alcohol	160 (92.3)	3 (1.7)	10 (5.8)
Driving 30 min after consuming two alcoholic drinks	145 (83.8)	11 (6.4)	17(9.8)
Driving after using marijuana	143 (82.7)	12(6.9)	18 (10.4)
Driving after using illegal drugs	156 (90.2)	4 (2.3)	13 (7.5)
How likely you would stop a **family member** from practicing the following behaviors			
Driving when they are intoxicated from alcohol	155 (89.6)	6 (3.5)	12 (6.9)
Driving 30 min after consuming two alcoholic drinks	141 (82.0)	16 (9.3)	15 (8.7)
Driving after using marijuana	144 (83.7)	8 (4.7)	20 (11.6)
Driving after using illegal drugs	155 (90.1)	6 (3.5)	11 (6.4)

Note: “Likely” and “Very Likely” responses were combined; “Very Unlikely” and “Not-Likely” were also combined.

## Data Availability

The data presented in this study are available on request from the corresponding author. The data are not publicly available due to ethical reasons.
